# Optimizing the mobility of residents with dementia: a pilot study promoting healthcare aide uptake of a simple mobility innovation in diverse nursing home settings

**DOI:** 10.1186/1471-2318-13-110

**Published:** 2013-10-18

**Authors:** Susan E Slaughter, Carole A Estabrooks

**Affiliations:** 1 Edmonton Clinic Health Academy, Faculty of Nursing, University of Alberta, Edmonton, Alberta, Canada

**Keywords:** Mobility, Knowledge translation, Healthcare aides, Context, Long-term care, Nursing home

## Abstract

**Background:**

Almost 90 percent of nursing home residents have some type of mobility limitation. Many spend most of their waking hours lying in bed or sitting. Such inactivity can negatively affect residents’ health and general well-being. This pilot study aimed to assess (1) the effect of the sit-to-stand activity on mobility outcomes of nursing home residents, (2) the effect of an audit-and-feedback intervention on uptake of the sit-to-stand activity by healthcare aides, and (3) the contextual factors influencing uptake of the sit-to-stand activity by healthcare aides.

**Methods:**

This quasi-experimental pilot study was conducted in two nursing homes in western Canada. Twenty-six residents with dementia completed the sit-to-stand activity with 56 healthcare aides during daily care; separately, 71 healthcare aides completed a research use and context survey. Preliminary mobility feedback was presented to healthcare aides in one site. Resident mobility was measured using the 30-second sit-to-stand test. Healthcare aide uptake of the activity was measured using documentation flowsheets and a survey-based measure. Context was measured using the Alberta Context Tool. Mobility and uptake outcomes were analyzed over time and by site with analysis of covariance. Spearman and Pearson correlations were used to correlate context data with research use.

**Results:**

Residents who more frequently completed the sit-to-stand activity were more likely to maintain or improve mobility compared with those who completed it less frequently (F=4.46; p=0.046, after adjustment for age). Uptake for one site was significantly different from the other (t-score=2.67; p=0.01, after adjustment for resident covariates). The audit-and-feedback intervention was associated with increased uptake of the activity from pre-intervention to post-intervention (t-score=-2.48; p=0.02). More context domains correlated significantly with aides’ use of conceptual research and information sources in one site than the other.

**Conclusions:**

The sit-to-stand activity is a promising means to maintain or improve transfer ability of nursing home residents with dementia. In the nursing home with initially weak uptake, strengthened uptake followed an audit-and-feedback intervention. Activity participation was higher in the site with stronger correlations between context and measured research use. Results are sufficiently promising to warrant proceeding with a full clinical trial.

## Background

Almost 90 percent of nursing home residents have some type of mobility limitation [1] which can negatively affect their health and general well-being. When residents’ mobility is compromised, not only do they experience difficulties performing daily activities such as walking, toileting, and socializing, but they are also prone to falls, pressure ulcers, incontinence, and pneumonia - all of which can lead to complications and hospitalization [2-6]. Despite evidence that low-intensity exercise can improve physical performance [7,8] and activities of daily living [8,9] among frail older adults in long-term care facilities, residents still spend the majority of their waking hours lying in bed or sitting [10,11].

**Figure 1:**
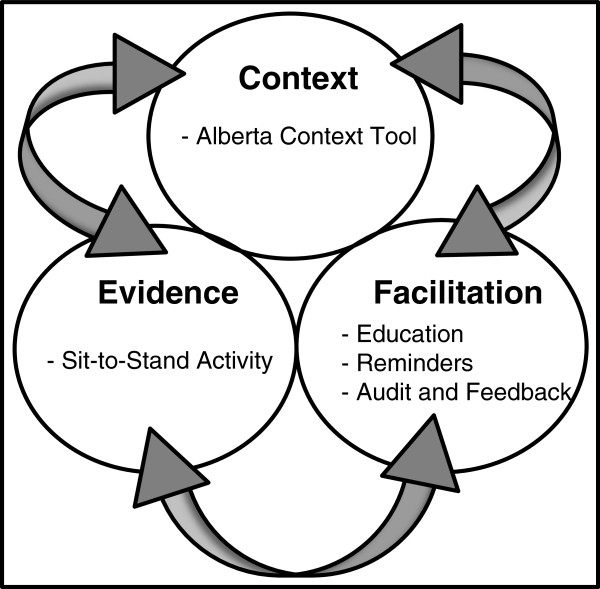
**The *promoting action on research implementation in health services *** **conceptual framework.**

The translation of research evidence into practice is slow, if it happens at all. For example, the proportion of care for older adults that is based on research has been estimated at 29% for urinary incontinence, 35% for cognitive impairment, and 34% for falls and mobility disorders [12]. The *Promoting Action on Research Implementation in Health Services* (PARIHS) framework, which guided this study, suggests that successful implementation of evidence into practice involves an interaction among robust evidence, strong facilitation, and a favorable context [13] (Figure 1). This pilot study examined each of these three aspects of successful implementation: the effect of introducing evidence (sit-to-stand activity) on nursing home resident outcomes, the effect of a method of facilitation (education, reminders, audit-and-feedback) on the uptake of the evidence by healthcare aides, and the effect of the contextual quality (Alberta Context Tool) associated with uptake of the evidence (see Figure 1).

### The evidence

Preliminary evidence suggests that performance of the sit-to-stand activity—as one component of an exercise program—may delay the trajectory of functional decline in long-term care residents [14-18]. However, these studies do not assess the value of the sit-to-stand as a single activity. The sit-to-stand activity is thought to be one of the most mechanically demanding for nursing home residents [19]. It usefully builds upon the basic function of transferring, which is fundamental to most basic activities of daily living [20,21]. Furthermore, the sit-to-stand activity possesses several of the attributes of an innovation that is more likely to be adopted, according to Rogers’ *Diffusion of Innovations* theory: low complexity, relative advantage, compatibility, and trialability [22].

### The facilitation

Although healthcare aides provide most of the direct care for residents in long-term care settings, few strategies intended to facilitate the implementation of evidence into practice have been developed with healthcare aides in mind [23]. Even less research has been conducted on the effect of *knowledge translation interventions* on innovation uptake in long-term care settings where healthcare aides work. Reviews on the dissemination and implementation of guidelines found that only 3% of studies were based in nursing homes [24,25]. Several knowledge translation interventions have been identified as potentially suitable for application to the nursing home setting to increase innovation uptake [26]. One such knowledge translation intervention is audit-and-feedback [27], for which modest evidence exists of success in increasing innovation uptake. Audit-and-feedback monitors the performance of health providers over time relative to a practice change, then reports back to them on their performance. Particularly relevant to our research, audit-and-feedback interventions have some documented success in increasing innovation uptake among healthcare aides [28,29].

### The context

Context is defined as the environment in which people receive healthcare services, and in which a proposed change is to be implemented [30]. Previous research has conceptualized innovation adoption as a discrete decision by individuals and focused on an outcome of interest. However, systematic reviews on organizational context emphasize the importance of context in understanding how and why innovations are adopted and assimilated into clinical practice [31]. The context in which an innovation is situated is as important a determinant of adoption and sustainability of the innovation as the innovation itself [31,32].

The purpose of this study was to assess the effect of the sit-to-stand activity (evidence) on the mobility outcomes of nursing home residents, the effect of an audit-and-feedback intervention (facilitation) on the uptake of the sit-to-stand activity by healthcare aides, and the contextual factors influencing the uptake of the activity (context).

## Methods

This quasi-experimental pilot study conducted in a western Canadian city was designed to answer the following research questions:

– 1) Does the sit-to-stand mobility innovation maintain or improve resident mobility?

– 2) How does an audit-and-feedback intervention influence uptake of the sit-to-stand innovation by healthcare aides?

– 3) What is the relationship between the contextual features of long-term care facilities and the uptake of the sit-to-stand mobility innovation by healthcare aides in those facilities?

**Figure 2:**
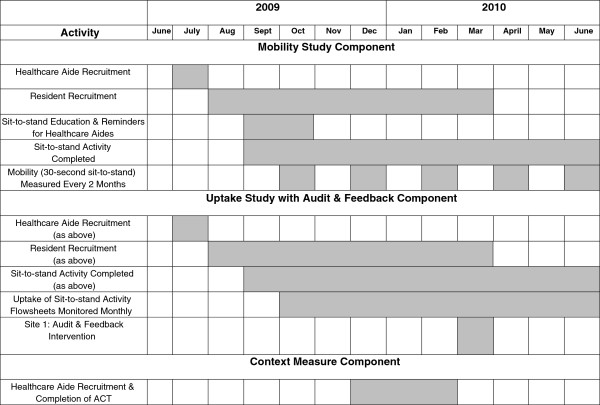
Project timeline and work plan for each study component.

Each of the three research questions was answered through a specific component of the pilot study. The first component assessed resident mobility outcomes before and after exposure to the sit-to-stand activity. The second component assessed the uptake or extent to which healthcare aides and residents completed the sit-to-stand activity in two different nursing home settings, and the relationship between uptake and an audit-and-feedback intervention. The third component examined the relationship between the contextual features of the two nursing homes and the uptake of evidence-based research. Figure 2 outlines the work plan for each study component in relation to the other components.

### Inclusion and exclusion criteria

From August 2009 to April 2010 we recruited a convenience sample of residents and healthcare aides from two purposively sampled nursing homes that were also participating in a larger study about organizational context [33]. Residents with Alzheimer’s disease and related dementia (as noted in their health record) were included in the study because these types of dementia are common among nursing homes residents and lead to a loss of mobility as the dementia progresses. Residents who were able to transfer independently or with the assistance of one person at baseline were eligible to participate. They did not have to speak English. Those with a serious physical illness or a life expectancy of less than six months at the time of recruitment were excluded from the study. Healthcare aides working directly with the participating residents were invited to participate. Nursing homes were selected for maximum variation (e.g., rural and urban, for-profit and public).

### Mobility innovation

The sit-to-stand activity involved a cooperative effort between the healthcare aides and the residents. Healthcare aides encouraged residents to slowly stand up and sit down as many times as possible on two occasions each day and evening shift. The sit-to-stand activity was carried out as part of the resident’s usual activities of daily living such as toileting or dressing. No time limit was placed on the duration of the activity. The number of sit-to-stand repetitions on a given occasion was individualized according to the resident’s abilities and fatigue.

### Mobility outcome measure

The sit-to-stand manoeuver is a functional activity that has been incorporated into a number of mobility measures including the time for five sit-to-stands and the number of sit-to-stands completed in 30 seconds (the 30-second sit-to-stand test). The 30-second sit-to-stand test was selected for this pilot study because residents in long-term care may be unable to complete more than two or three sit-to-stands [10,14,34-36]. Resident mobility was measured, using the 30-second sit-to-stand test, by a single research assistant when each participating resident was recruited to the study and every two months thereafter until the end of the study. Using a stopwatch, the research assistant instructed resident participants to stand up and sit down as many times as possible from a standard armchair until they were asked to stop after 30 seconds. In community-dwelling older adults, this mobility measure shows test-retest reliability (r = 0.89 with testing two to five days apart); criterion validity (r = 0.77 chair stand performance compared with leg press performance); and discriminant validity (three age groups differed p < .01; high & low activity groups differed p < .001) [34].

### Knowledge translation interventions

At the beginning of the study one of the investigators (SS) introduced healthcare aides to the sit-to-stand activity during 15-minute education sessions, which included a description of the potential benefits of the sit-to-stand activity for the residents and healthcare aides, a demonstration of the activity and related documentation, and an explanation of the reminders that identified the residents participating in the study. Approximately four education sessions were conducted in Site 1 and eight sessions in Site 2 to reach as many healthcare aides as possible. The education sessions were typically conducted following the change of shift meeting (day to evening) and included three to five healthcare aides in each session. Initially, paper-based reminders (bedside stickers and a conference room poster) were introduced to both sites. Towards the end of the study, in March 2010, an audit-and-feedback intervention was introduced to Site 1 when minimal participation in the sit-to-stand activity became apparent. In the audit-and-feedback intervention, the researchers summarized preliminary resident mobility outcome data in a poster. The poster data were verbally presented in 10 minutes to the Director of Care and subsequently, on two occasions, to healthcare aides and other unit staff at the change of shift meeting (day to evening).

### Uptake measure

Uptake was measured in two ways. First, in consultation with nurse managers, we integrated documentation of resident performance of the sit-to-stand activity into the existing health record flowsheet completed by healthcare aides. Healthcare aides recorded on this flowsheet the number of sit-to-stands that the resident completed on each of two occasions on the day shift and on the evening shift (i.e., four occasions per day). A research assistant scored the flowsheet for each occasion, with a score of 1 denoting a completed occasion of sit-to-stand activity and 0 denoting that the sit-to-stand activity was not completed. The number of occasions per month that each resident participated in the sit-to-stand activity was a measure of the mobility innovation’s uptake. Second, as part of the larger study which took place concurrently in the two sites [33], we measured uptake through healthcare aide responses to an interview-based survey including questions pertaining to aides’ use of conceptual research (5 items [37]) and information sources (10 items).

### Context measure

We measured the organizational context in each facility using the Alberta Context Tool, a 56-item survey instrument consisting of eight dimensions: leadership (6 items), culture (6 items), evaluation (6 items), social capital (6 items), informal interactions (7 items), formal interactions (5 items), structural and electronic resources (11 items), and organizational slack (9 items representing three sub-concepts - staff, space, and time) [38]. The Alberta Context Tool is a reliable and valid measure of context that is designed to be completed by individual care providers. It discriminates between the contextual features of pediatric units [38,39] and can also assess the organizational context of nursing homes when completed by healthcare aides [40]. The Alberta Context Tool was completed by a sample of healthcare aides at our two study sites as part of a larger concurrent study [33]. Healthcare aides completing the Alberta Context Tool were not necessarily the same aides participating in the sit-to-stand and uptake interventions.

### Sample characteristics

Resident participant characteristics were derived from the Resident Assessment Instrument – Minimum Data Set (version 2.0) [41] in residents’ health records. These included age and scores on the Cognitive Performance Scale (CPS) [42,43], the Depression Rating Scale (DRS) [44], and the Changes in Health, End-stage disease and Symptoms and Signs (CHESS) [45]. The monthly incidence of resident falls was monitored for three months before and throughout the study using data from the managers’ falls logs at each site. Characteristics of healthcare aide participants were collected when the Alberta Context Tool was administered. The characteristics of these healthcare aides were extrapolated to the aides participating in the sit-to-stand activity because the two studies took place concurrently. Many of the healthcare aides would have participated in both studies, such that the characteristics of those in one study would be a good approximation of the characteristics of those in the other. These data included age, education and employment history. Characteristics of participating facilities included ownership model (public or for-profit), setting (urban or rural), age, and number of beds.

### Data analysis

#### Research question 1: evidence

Resident mobility scores on the 30-second sit-to-stand test were summarized using descriptive statistics. Resident mobility outcomes were assessed by comparing the change in their scores on the 30-second sit-to-stand test from early December 2009 to early February 2010. A two-month period was expected to provide the residents enough exposure to the sit-to-stand activity with healthcare aides to produce a detectable change in their mobility. This particular two-month period coincides with the time when healthcare aides and the majority of residents were recruited to the study and were at ease completing the sit-to-stand activity. It also coincides with the time when data from the Alberta Context Tool were collected. An analysis of covariance (ANCOVA) compared change in mobility (dependent variable) with the extent that residents regularly completed the sit-to-stand activity with healthcare aides (high dose vs low dose) during December and January. High dose was defined as completing the activity on two occasions per day or more (≥ 120 occasions over two months). Low dose was defined as completing the activity fewer than two occasions per day (< 120 occasions over two months). This cut point of 120 occasions over two months was half of the study target of four occasions per day. In the ANCOVA, we adjusted for resident covariates of age, CPS, DRS and CHESS.

#### Research question 2: facilitation

Using a two-way ANCOVA we assessed the interaction effect of site by time on the uptake of the sit-to-stand activity for December 2009, February 2010 and April 2010. This included the period when the audit-and-feedback intervention was introduced to Site 1 (in March 2010). We adjusted for the resident covariates of age, CPS, DRS and CHESS.

#### Research question 3: context

Characteristics of participating residents, healthcare aides and facilities for each site were summarized using descriptive statistics. We assessed the relationship between uptake and context using inferential statistics to compare Site 1 with Site 2 in two ways. Firstly we examined the main effect of site on uptake using the two-way repeated measures ANCOVA described above. Secondly the eight domains from the Alberta Context Tool for each site were summarized using descriptive statistics. Pearson and Spearman’s rho correlations between each domain of the Alberta Context Tool and the two measures of research uptake [i.e., the measure of aides’ use of conceptual research and information sources] were computed for each facility as appropriate. Data were analyzed using STATA 10 (StataCorp, 2007, College Station, TX: Stata Corporation).

### Ethics

This study was approved by the Health Research Ethics Board of the University of Alberta. With the support of senior administrators in the two nursing homes, we recruited residents and healthcare aides to the study. Since residents did not have the capacity to provide informed consent, written informed consent was obtained from the authorized representatives of all resident participants [46]. Unit managers or designates approached substitute decision makers/authorized representatives of eligible residents, using a standard script for permission to provide rpresentatives’ contact information to the researchers. The research assistant then contacted the authorized representative for consent. Residents’ assent to participate in the study was evaluated by their willingness to cooperate with the baseline mobility measurement [47]. The research assistant obtained informed written consent from healthcare aides during pre-arranged unit meetings.

## Results

Over a nine-month period, from July 2009 to March 2010, a total of 45 residents (61% response rate) and 56 healthcare aides (57% response rate) were recruited to carry out the sit-to-stand activity in two nursing homes. During the period from December 2009 to February 2010, 26 residents completed the sit-to-stand activity with the 56 healthcare aides. Of the 45 residents recruited to the study, 19 were not included in the analysis of the mobility outcome because: 5 were recruited after February, 5 dissented to participate after initially assenting, 4 lost mobility and could no longer participate in the activity because they required a two-person transfer assist, 3 died before February and 2 became too cognitively impaired to follow instructions for the 30-second sit-to-stand test. Compared to the 11 participating residents in Site 1, the 15 participating residents in Site 2 had significantly more health instability as measured by the CHESS (p = 0.004), were more depressed as measured by the DRS (p = 0.04), and tended to be more cognitively impaired.

During this same period from December 2009 to February 2010, 71 healthcare aides completed the Alberta Context Tool as part of a larger study. Since the timing of data collection for these two study components coincided, many of the 56 healthcare aides completing the sit-to-stand activity were included in the group of 71 healthcare aides completing the Alberta Context Tool.

Table 1 summarizes the characteristics of the 26 residents who completed the sit-to-stand activity between December and January, and the 71 healthcare aides who completed the Alberta Context Tool during this same period. Significantly more healthcare aides in Site 2 had healthcare aide certificates (p = 0.004). Site 1 was an older (more than five years old), smaller, rural for-profit facility with fewer than 100 beds, while Site 2 was a newer (less than five years old), mid-sized, urban, public facility with between 100 and 150 beds.

**Table 1:** Resident and healthcare aide characteristics by nursing home site

**Characteristics**	**Site 1**	**Site 2**	**p**-**value**
Residents	(n=11)	(n=15)	
Age: Mean (SD)	84.6 (5.8)	86.8 (4.4)	0.27
Cognitive Performance Scale: Mean (SD)	2.73 (1.0)	3.40 (1.1)	0.12
Depression Rating Scale: Mean (SD)	2.64 (1.43)	4.27 (2.2)	0.04
Changes in Health, End-stage disease and Symptoms and Signs: Mean (SD)	0.64 (0.67)	1.86 (1.1)	0.004
Healthcare aides completing the Alberta Context Tool	(n=27)	(n=44)	
HCA age < 40 years: n (%)	13 (48%)	16 (36%)	0.24
Time on unit (months): Mean (SD)	44.2 (55.8)	27.7 (21.2)	0.08
High school education: n (%)	20 (74%)	39 (89%)	0.11
Healthcare aide certificate: n (%)^a^	17 (63%)	40 (91%)	0.004

^a^Pearson Chi square.

### Question 1: evidence

Table 2 reports mean resident scores on the 30-second sit-to-stand outcome measure in early December 2009 and early February 2010, in relation to the extent to which residents completed the activity with healthcare aides, during December 2009 and January 2010. Residents in the high dose group (≥ 120 occasions in December and January) had an average increase of two sit-to-stands completed in 30 seconds compared with a decrease of two sit-to-stands completed for those in the low dose group (< 120 occasions in December and January). Table 3 summarizes the ANCOVA for the 30-second sit-to-stand outcome measure, comparing residents receiving a high dose (≥ 120 occasions) with those receiving a low dose (< 120 occasions) of the mobility innovation over two months (from December 2009 to February 2010) after adjusting for age (F=4.46; p = 0.046). No statistical differences were evident between the doses of activity when adjusting for cognition (p = 0.57), depression (p = 0.11) and medical stability (p = 0.84). Note that only three residents, all from Site 2, were in the high dose group; the remaining 23 residents were in the low dose group.

**Table 2:** **Mean 30**-**second sit**-**to**-**stand score per month by dose of activity**

**30-second sit-to-stand test averages, December 2009 to February 2010 (n=26)**			
**Activity dose**	**December 2009 M (SE)**	**February 2010 M (SE)**	**Difference**
Low Dose	7.26 (.88)	5.35 (.69)	-1.91
High Dose	4.00 (1.00)	6.00 (1.53)	2.00

SE = standard error.

**Table 3:** **Analysis of covariance**: **resident mobility change from December to February by dose of activity with healthcare aides** (**high vs**. **low**)

**Variables**	**F-score**	**p-value**
Dose	4.46	.046
Age	3.16	.089

High dose ≥ 120 occasions.

Low dose < 120 occasions.

### Question 2: facilitation

**Figure 3:**
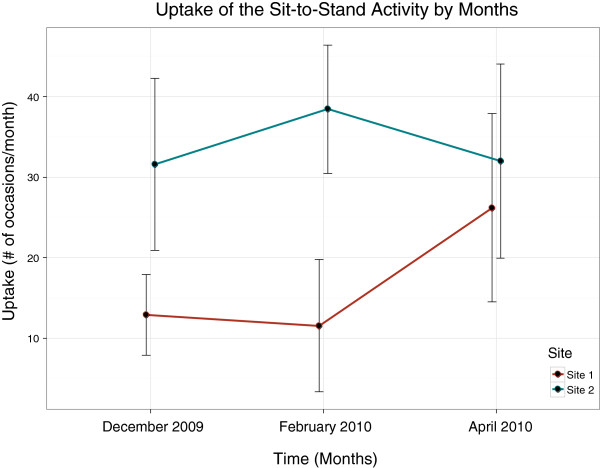
**Mean uptake of sit-to-stand activity with 95% confidence intervals by time and site.** Note: audit-and-feedback intervention introduced to Site 1 in March.

Table 4 reports the means for uptake of the sit-to-stand activity. In December 2009 the range in the number of occasions of completing the sit-to-stand activity in Site 1 was 0 to 40 while the range in Site 2 was 6 to 75. Results of the two-way analysis of covariance are reported in Table 5. To summarize, a significant main effect for time (p=0.01) indicates a change in uptake over time. As well, a significant main effect for site (p=0.01) indicates that the uptake for site 1 was significantly different than the uptake for site 2. There was a significant interaction effect such that the site 1 mean uptake increased over time from 12.9 to 26.2 occasions, while the site 2 mean uptake stayed relatively constant over time from 31.6 to 32 occasions (p=0.02). The covariates age (p=0.16), CHESS (p=0.95), CPS (p=0.65) and DRS (p=0.92) did not have a significant influence on the findings. The changes in uptake over time by site are graphically displayed in Figure 3.

**Table 4:** Mean number of occasions of activity per month by site

**Site 1 mean (SE)**			**Site 2 mean (SE)**		
**December '09 n = 16**	**February '10 n = 11**	**April '10 n = 11**	**December '09 n = 21**	**February '10 n = 18**	**April '10 n = 16**
12.88 (2.55)	11.54 (4.20)	26.18 (5.97)	31.57 (5.45)	38.44 (4.06)	32.00 (6.16)

SE = standard error.

**Table 5:** **Two**-**way analysis of covariance**: **uptake of the activity change from December to February to April between site 1 and site 2**

**Variables**	**t-score**	**p-value**
Time	2.56	0.01
Site	2.67	0.01
Interaction (site x time)	-2.48	0.02
Age	-1.43	0.16
CPS	0.46	0.65
DRS	-0.10	0.92
CHESS	-0.06	0.95

### Question 3: context

Mean scores of the eight domains of the Alberta Context Tool are summarized in Table 6. Site 2 scored higher on almost all the context domains; however, the differences only reached statistical significance for the evaluation domain. Correlations between the domains of the Alberta Context Tool and two measures of research uptake were compared for each facility (see Table 7). More of the context domains of Site 2 correlated significantly with aides’ use of conceptual research and information sources compared with Site 1. Context domains with significant correlations for site 2, but not for site 1, included culture, evaluation, formal interactions, structural resources and organizational slack – space.

**Table 6:** Alberta context tool and research use scores by nursing home site

**Scores on Alberta context tool derived scales: mean (SD)**			
**Domains**	**Site 1 (n=25)**^**†**^	**Site 2 (n=43)**	**p-value**
Leadership^b^	3.63 (.58)	3.86 (.66)	.163
Culture^b^	3.66 (.63)	3.82 (.57)	.279
Evaluation^b^	3.41 (.66)	3.00 (1.02)	.047^§^
Formal Interactions^a^	N/A	N/A	.491
Informal Interactions^a^	N/A	N/A	.657
Social Capital^b^	3.97 (.48)	4.06 (.45)	.412
Structural Resources^a^	N/A	N/A	.125
Organizational Slack - Staff^b^	2.21 (1.17)	2.49 (1.06)	.326
- Space^b^	2.84 (1.23)	2.92 (1.20)	.798
- Time^b^	2.75 (.65)	2.75 (.73)	1.000
Research Use Derived Scales: Mean (SD)			
Conceptual Research Use^b^	3.51 (.80)	3.67 (.70)	.386
Information Sources^a^	N/A	N/A	.668

^†^Changes in sample size from totals in Table 1 due to missing data.

^a^Wilcoxon rank-sum test.

^b^Two group mean comparison t-test.

^§^Equal variance not assumed.

N/A = not applicable: means are not applicable for ordinal data.

**Table 7:** **Spearman**’**s rho correlations between context and research use by nursing home site**

**Alberta context tool domains**	**Site 1 correlations (p) (n=26)**		**Site 2 correlations (p) (n=44)**	
	**Conceptual research use**^**‡**^	**Information sources**	**Conceptual research use**^**‡**^	**Information sources**
Leadership^‡^	.59 (.002)	.41 (.04)	.29 (.06)	.20 (.20)
Culture^‡^	.30 (.14)	.04 (.86)	.35 (.02)	.34 (.02)
Evaluation^‡^	.22 (.28)	-.03 (.90)	.32 (.03)	.30 (.048)
Formal Interactions	.30 (.13)	.10 (.65)	.14 (.37)	.35 (.02)
Informal Interactions	.23 (.27)	.40 (.04)	-.02 (.89)	.11 (.50)
Social Capital^‡^	.46 (.02)	.10 (.61)	.34 (.03)	.18 (.26)
Structural Resources	.05 (.81)	.18 (.38)	.30 (.045)	.44 (.003)
Organizational Slack - Staff^‡^	.32 (.12)	.20 (.33)	.14 (.38)	.22 (.15)
- Space^‡^	-.36 (.07)	.05 (.79)	.33 (.03)	.48 (.0009)
- Time^‡^	.34 (.09)	.45 (.02)	.16 (.30)	.50 (.0006)

Spearman’s rho correlations were computed for variables with count data.

^‡^Pearson’s correlations were computed for variables with continuous data.

### Safety

During the study, four (22%) residents fell in Site 1 and 20 (80%) residents fell in Site 2. None of these falls led to serious injury, and none occurred while carrying out the sit-to-stand activity. Of the 45 resident participants, eight stopped falling after they entered the study, seven started to fall after entering the study, and the fall status of the remaining residents did not change after they entered the study. These three fall categories did not differ between the two nursing homes (*χ*^*2*^ = 1.98; p = 0.37).

## Discussion

This pilot study provides evidence that the sit-to-stand activity can safely maintain, and in some cases modestly improve, residents’ ability to stand from a chair. The findings also show that introducing an audit-and-feedback knowledge translation intervention to healthcare aides was associated with an increased level of sit-to-stand activity uptake in Site 1 where initial uptake was weak. Additionally, the data suggest that the higher scores for nursing home context in Site 2 are correlated with improved research uptake scores and were associated with improved uptake of the sit-to-stand activity. In this pilot study the PARIHS framework provided useful structure (evidence, facilitation and context) to guide the development of the research questions, the organization of the data collection and the presentation of the findings [13].

Barriers to engaging in activity are present at any age but especially for older adults living in long-term care settings. Other studies have investigated the sit-to-stand activity as one element of an exercise program in community-based settings [48-50] or have introduced an exercise through the use of additional therapists or research assistants. This pilot study makes a unique contribution to the literature with an investigation of the sit-to-stand activity as a feasible stand-alone activity integrated into existing care routines and implemented by existing healthcare aide staff.

This study diverges from the mobility research literature in three other important ways. First, it monitored the fidelity of the uptake of the sit-to-stand mobility innovation. Second, it introduced an audit-and-feedback knowledge translation intervention to respond to weak uptake of the mobility innovation. Third, it examined how the contextual factors in two long-term care facilities were associated with uptake of the mobility innovation. To our knowledge, no other studies examine mobility outcomes of residents with dementia and the intermediate adoption outcomes of care providers against the backdrop of an audit-and-feedback intervention and contextual factors in long-term care settings.

This pilot study has positive implications for practice in three areas. First, the sit-to-stand activity turns a necessary, everyday movement into a repetitive and parsimonious activity that healthcare aides are able to integrate into residents’ daily routines, amidst the fiscal and time constraints facing many nursing homes. Second, the study demonstrates that an audit-and-feedback knowledge translation intervention can lead to strengthened uptake of a mobility innovation in an initially unreceptive nursing home. Others have suggested that an audit-and-feedback intervention can have a modest but positive influence on the uptake of an evidence-based practice [51,52], especially when initial uptake is low [27]. This leads to the third practice implication; our study highlights an association between context and uptake. After adjusting for resident characteristics, uptake of the sit-to-stand activity was significantly higher in Site 2 compared with Site 1. Site 2 was also the site that had more significant correlations between the domains of the Alberta Context Tool and the measure of research use by aides. Contextual differences across nursing homes internationally [53], and even across units within a nursing home [39], can influence the manner in which aides, nurses and managers provide care [53]. Understanding how the uptake of innovation varies across context is increasingly recognized to be important in the spread of innovations [54].

Understanding the specific contextual factors that are likely to lead to high or low uptake of any innovation may help to identify where knowledge translation interventions could be useful to promote uptake. For example, in this pilot study, despite the small sample size, Site 1 scored significantly higher than Site 2 on the evaluation domain of the Alberta Context Tool. Evaluation in this instrument is defined as “the process of using data to assess group/team performance and to achieve outcomes in organizations or units” [40]. Thus it is not surprising that with a high score on evaluation, Site 1 responded well to the audit-and-feedback intervention.

Resident safety was not compromised with the introduction of the sit-to-stand activity. This is consistent with the experience of others in carrying out exercise interventions in older adults with chronic disease [16,55-58]. In this pilot study, even the most vulnerable residents with dementia and significant comorbidities were able to safely complete the activity with healthcare aides. Although there was significant variation across residents in the number of occasions that the activity was completed per month (e.g., from 0 to 75 occasions), one of the important messages of this article is the significance of the facility or contextual factors that influenced the uptake of the sit-to-stand activity. Certainly individual level factors (both healthcare aide and resident factors) could have influenced participation (or uptake). It is well known that residents with dementia occasionally can be unwilling to respond to the prompting of healthcare aides. Resident refusal is a common occurrence during usual care when healthcare aides try to assist residents with dementia in completing activities of daily living. However, results of the two-way ANCOVA (Table 5) are an important demonstration that individual characteristics (covariates) of residents (age, cognition score, depression score and medical instability score) did not significantly affect activity uptake, whereas site context did significantly affect activity uptake.

The limitations associated with this pilot study are those common to pilot studies. These limitations include a small sample size, the absence of a control group and non-randomization. Nevertheless, this pilot project provided experience with the sit-to-stand activity to inform a full controlled clinical trial, currently under way [59]. Future research is indicated to refine an understanding of which contextual factors facilitate or inhibit the sit-to-stand mobility activity’s uptake into healthcare aides’ routines, as well as which knowledge translation interventions most efficiently lead to the adoption and sustainability of the mobility innovation. Moreover, this pilot study was conducted among a sample of residents living with dementia. Future research can determine whether this mobility innovation and related knowledge translation interventions addressing various contextual factors are transferable to other settings such as homecare and assisted living, where the older adult population may be more cognitively healthy.

## Conclusion

Optimizing the mobility of nursing home residents is a sleeping giant because so many nursing home residents have some type of mobility limitation. The potential benefits of maintaining residents’ mobility is understudied and have not been fully realized. Given that transfers are an essential component of most basic activities of daily living, supporting and maintaining this ability in the form of the sit-to-stand innovation represents an important opportunity to contribute to the function and well-being of older adults living in residential nursing homes. This is a particularly promising line of research because the sit-to-stand activity is a feasible, inexpensive innovation that can be worked into usual nursing home care routines [32]. Moreover, varying contextual factors were associated with varying degrees of uptake of the sit-to-stand innovation. Even in a setting where early uptake of the sit-to-stand activity was weak, however, the use of the audit-and-feedback knowledge translation intervention was able to strengthen uptake. These encouraging findings, in conjunction with the observed positive mobility outcomes and uptake of the mobility intervention, are strong support for further research.

## Abbreviations

CHESS: Changes in health, end-stage disease and symptoms and signs; CPS: Cognitive performance scale; DRS: Depression rating scale; ANCOVA: Analysis of covariance; SD: Standard deviation; SE: Standard error.

## Competing interests

The authors declare that they have no competing interests.

## Authors’ contributions

SES conceived of the project, designed the study and collected the data. CAE collaborated on the design of the study, interpretation of the data and writing of the article. All authors read and approved the final manuscript.

## Pre-publication history

The pre-publication history for this paper can be accessed here:

http://www.biomedcentral.com/1471-2318/13/110/prepub
